# Effect of Traditional Chinese Medicine on Inflammatory Mediators in Pediatric Asthma

**DOI:** 10.1155/2016/5143703

**Published:** 2016-06-09

**Authors:** Hui Du, Yonghong Wang, Yumin Shi, Jian Yu, Wen Sun, Yiqun Zhang

**Affiliations:** Department of Traditional Chinese Medicine, Children's Hospital of Fudan University, No. 399, Wanyuan Road, Shanghai 201102, China

## Abstract

*Objective*. To observe the effects of empirical prescriptions of traditional Chinese medicine (TCM) on inflammatory mediators in pediatric asthma and to explore the underlying molecular mechanism in the treatment of asthma.* Methods*. A total of 182 children with asthma were randomly placed into either the TCM group (*n* = 97) or the salbutamol and montelukast (SM) group (*n* = 85). Patients in the TCM group were treated with a series of empirical prescriptions of TCM, while those in the SM group received salbutamol and montelukast. Both groups received their respective treatment for 12 weeks. There were 35 patients in TCM group and 34 patients in SM group providing venous blood. Real-time PCR was used to determine the mRNA expression levels of interleukin- (IL-) 10, IL-17, matrix metalloproteinase-9 (MMP-9), and transforming growth factor *β*1 (TGF-*β*1) in peripheral blood mononuclear cells before and after treatment. Enzyme-linked immunosorbent assay was used to measure the levels of IL-10, IL-17, MMP-9, and TGF-*β*1 in peripheral blood before and after treatment.* Results*. The mRNA expression of TGF-*β*1 in the SM group was downregulated (*P* = 0.00) after treatment. No significant differences were found between the TCM group and the SM group after treatment (*P* > 0.05). In the TCM group, the levels of IL-10, IL-17, and MMP-9 significantly decreased after treatment (*P* = 0.01, 0.04, and 0.03, resp.). In the SM group, IL-17, MMP-9, and TGF-*β*1 levels significantly decreased after treatment (*P* = 0.00, 0.03, and 0.00, resp.). There was no significant difference between the two groups regarding the levels of IL-10, IL-17, TGF-*β*1, and MMP-9 (*P* > 0.05). The difference of the level of IL-17 was negatively correlated with the change of C-ACT score in TCM group and SM group.* Conclusion*. TCM has a regulatory effect on the balance of some inflammatory mediators in pediatric asthma.

## 1. Introduction

Asthma is a heterogeneous disease that is usually characterized by chronic airway inflammation. It involves participation of eosinophil granulocytes, T cells, mast cells, and cytokines. Asthma is the most common chronic childhood disease and the leading cause of childhood morbidity from chronic disease [1]. Although the pathogenesis of asthma is not yet clear, it is believed that an imbalanced ratio of Th1 and Th2 cell function is an important mechanism of this disease [2]. In addition, type 17 helper (Th17) cells play an important role in the development of asthma [3]. Studies showed that matrix metalloproteinase-9 (MMP-9) and transforming growth factor-beta 1 (TGF-*β*1) are also closely related to airway inflammation and airway remodeling [4, 5].

Traditional Chinese medicine (TCM) treatments for asthma have achieved good results. A previous study showed that empirical prescriptions of TCM effectively control asthma exacerbation and improve lung function [6]. However, the mechanism of the empirical prescriptions is unclear. This study was designed to explore the regulatory effect of empirical prescriptions of TCM on the inflammatory mediators and cytokines related to asthma in children in order to provide a theoretical basis for clinical practice.

## 2. Materials and Methods

### 2.1. Study Design

This study was a single-blind randomized controlled trial conducted at the Children's Hospital of Fudan University. Conventional treatment (salbutamol and montelukast) for asthma constituted the positive control treatment for the determination of the clinical effect of TCM treatment regimen on pediatric asthma. This study obtained approval from the Ethics Committee of Children's Hospital of Fudan University (number [2013] 065) and registered at http://clinicaltrials.gov/ct2/show/NCT02341573.

### 2.2. Inclusion and Exclusion Criteria

This study followed diagnostic criteria established in 2008, in accordance with the “standards in the Guideline of Childhood Bronchial Asthma” by the Pulmonology Group of the Pediatric Branch of the Chinese Medical Association [7]. TCM syndrome was defined according to “standards for the diagnosis and clinical effects on TCM syndrome” by the State Administration of Traditional Medicine [8]. Patients aged 3–12 years old with a syndrome meeting the above criteria for the diagnosis of pediatric asthma and cough-variant asthma were enrolled into the study. Asthmatic episodes were catalogued according to TCM syndrome differentiation as follows: a form of phlegm-heat obstructing the lungs or cold fluid retention in the lungs, lung, and spleen deficiencies or deficiency of kidneys in clinical remission stage of asthma. Patients with congenital diseases of the respiratory system, tumors, immunodeficiency diseases, or cardiovascular diseases were excluded.

### 2.3. Subjects

A total of 182 children with asthma were recruited between March 2013 and December 2014. They were randomly placed (using the random number table method) into either the SM (salbutamol and montelukast) group (*n* = 85) or the TCM group (*n* = 97). All the children who signed the consent and were willing to provide venous blood were enrolled and then randomly assigned to separate groups. At the end of this trial, there were 35 patients in TCM group and 34 patients in SM group providing venous blood before and after treatment. There were 17 girls and 18 boys with a mean age of 5.0 ± 1.6 years and a mean disease course of 8.3 ± 6.4 months in the TCM group. In SM group, there were 15 girls and 20 boys with a mean age of 5.0 ± 1.2 years and a mean disease course of 7.6 ± 6.5 months; one girl was lost to follow-up. There were no significant differences between two groups regarding age, gender, and disease course.

### 2.4. Interventions

*TCM Group*. Patients received a series of empirical TCM-oriented treatments. The initial treatment was Shegan Mixture (hospital preparations, Shanghai Liantang Pharmaceutical Co., batch number 20130101) corresponding to the patients in acute exacerbation stage of asthma. The composition of this prescription was as follows: 9 g ephedrine, 9 g almond, 6 g* Belamcanda chinensis*, 9 g* Rorippa indica*, 9 g* Radix scutellariae*, and 9 g batryticated silkworm. It was administered three times daily in a 10 mL mixture, over a 7 d treatment course. At follow-up visits during the fourth and eighth weeks, the prescription was adjusted according to TCM syndrome and SC scores. Patients in chronic persistent stage of asthma were treated with Shegan Mixture and Astragalus kidney tonic mixture. Patients in clinical remission were treated with Astragalus kidney tonic mixture comprised of 9 g Astragalus, 9 g atractylodes, 15 g Chinese yam, 9 g* Radix pseudostellariae*, 12 g* Poria cocos*, and 9 g medicinal Indian mulberry. Complementary herbs were added or removed according to presenting symptoms. For example, magnolia flower and* Fructus xanthii* were administered for nasal congestion and sneezing, ephedrine root and floating wheat were administered for hyperhidrosis, and* Semen raphani* and* F. forsythia* were prescribed for dry feces. These treatments were administered for 12 weeks.

The criteria for the quality of the herbs used were in accordance with the Chinese pharmacopeia (2005) [9].

*SM Group*. Patients with acute exacerbation of asthma were mainly treated with bronchial relaxants, salbutamol sulfate sustained release capsules (Ethypharm Pharmaceutical Co., Ltd., Shanghai, China, batch number 121201), 2–4 mg twice daily for 7 d. Patients in clinical remission were treated with leukotriene receptor antagonists, montelukast sodium chewable tablets (MSD pharmaceutical Co., Ltd., batch number 120054), 4-5 mg nightly for 3 months. Patients with chronic persistence of asthma were treated with salbutamol and montelukast.

Antibiotics or atomization treatment was administered in cases of acute bacterial infection and severe wheezing.

### 2.5. Outcomes

The outcomes were changes in the levels of IL-10, IL-17, MMP-9, and TGF-*β*1 in peripheral blood, and the mRNA expression levels of those cytokines in peripheral blood mononuclear cells (PBMCs) before and after 12 weeks of treatment, as well as changes in children asthma control test (C-ACT) scores before and after 12 weeks of treatment.

#### 2.5.1. C-ACT

C-ACT is a widely used asthma control assessment tool that is recognized by the Global Initiative for Asthma (GINA). This test includes seven questions and is divided into two parts: the first four questions are self-administered by the child; the last three questions are completed by a caregiver on the basis of the child's condition during the previous 4 weeks. With a total of 27 points, scores of 23 to 27 are classified as well controlled; 20~22 as partly controlled; and 0~19 as very poorly controlled asthma. The quality of this test was highly controlled by specialist.

#### 2.5.2. Specimen Collection and Processing

Peripheral blood samples (2 mL) were taken aseptically from asthmatic children and injected into the EDTA anticoagulation tubes with gentle shaking. Plasma was separated by centrifugation for 5 min at a speed of 2500 rpm and frozen for later analysis. PBMCs were separated with the Ficoll method (Huajing Biological Engineering Co. Ltd., Shenzhen, China).

#### 2.5.3. Real-Time Polymerase Chain Reaction (Real-Time PCR)

Total RNA was extracted from PBMCs using Trizol RNA extraction reagent (Life Technologies, Carlsbad CA, USA) and reverse-transcribed into cDNA (PCR MasterMix kit, Toyobo Co., Ltd., Osaka, Japan). Primers of each gene were designed using Primer Premier 5.0 (PREMIER Biosoft, Palo Alto, CA, USA) according to the cDNA sequences obtained from the National Center for Biotechnology Information. The primer sequences and amplicon sizes are shown in Table 1. Quantitative real-time PCR (qRT-PCR) was performed using SYBR Green Premix Ex Taq in a Mastercycler ep realplex instrument (Eppendorf AG., Hamburg, Germany). The PCR protocol was as follows: predenaturation for 20 min at 95°C followed by denaturation for 30 s at 95°C, annealing for 30 s at 54-55°C, and extension for 40 s at 72°C for 40 cycles. The relative amount of target gene mRNA was normalized to GAPDH.

#### 2.5.4. Enzyme-Linked Immunosorbent Assey (ELISA)

Plasma was obtained by sample centrifugation and anticoagulation. The content of IL-10, IL-17, MMP-9, and TGF-*β*1 was detected with a double antibody sandwich enzyme-linked immunosorbent assay (ELISA) kit, according to the kit instructions. The kits were supplied by RayBiotech, Inc., American. Optical density (OD) values were detected with an enzyme-labeled meter (Perlong Medical Instrument Co., Ltd., Beijing, China) at a wavelength of 450 nm. The diluted concentration standards used for IL-10, IL-17, MMP-9, and TGF-*β*1 were 150, 75, 37.5, 18.75, 9.38, 4.69, 2.34, and 0 pg/mL, 6000, 3000, 1500, 750, 375, 187.5, 93.75, and 0 pg/mL, 6000, 2000, 666.7, 222.2, 74.07, 24.6, 8.23, and 0 pg/mL, and 60, 20, 6.667, 2.222, 0.741, 0.247, 0.0823, and 0 ng/mL, respectively.

### 2.6. Statistical Analysis

Intention-to-treat analysis was performed. Demographic data were analyzed to identify baseline equivalencies and differences between the two groups. Continuous variables were presented as mean ± standard deviation (SD), evaluated by using Student's *t*-tests based on normal distribution. Data presented as abnormal distribution were evaluated by using *t*-tests after log transformation. Correlation analysis of normal distribution variables was evaluated by using Spearman. All data were analyzed using SPSS version 19 software. *P* < 0.05 was considered statistically significant.

## 3. Results

### 3.1. Comparison of the mRNA Expression Levels of IL-10, IL-17, TGF-*β*1, and MMP-9

No significant difference was found between the TCM group and the SM group after treatment (*P* > 0.05) (Table 2). In the SM group, the mRNA expression of TGF-*β*1 was downregulated (*P* = 0.00) after treatment. There was no significant difference of the mRNA expression of IL-10, IL-17, TGF-*β*1, and MMP-9 after treatment in TCM group (Table 3).

### 3.2. Comparison of the Levels of IL-10, IL-17, TGF-*β*1, and MMP-9 in Peripheral Blood

There was no significant difference in the levels of IL-10, IL-17, TGF-*β*1, and MMP-9 in peripheral blood between the TCM group and the SM group after treatment (*P* > 0.05). In the TCM group, the levels of IL-10, IL-17, and MMP-9 were significantly decreased after treatment (*P* = 0.01, 0.04, and 0.03, resp.). In the SM group, IL-17, MMP-9, and TGF-*β*1 levels were significantly decreased after treatment (*P* = 0.00, 0.03, and 0.00, resp.) (Tables 4 and 5).

### 3.3. Correlation between C-ACT Score and the Levels of IL-10, IL-17, TGF-*β*1, and MMP-9

The TCM group showed significantly higher C-ACT scores after 12 weeks of treatment than those of patients before treatment (after 22.5 ± 1.9 versus before 16.5 ± 1.9, *P* < 0.001). In the SM group, C-ACT scores significantly increased after 12 weeks of treatment (after 22.1 ± 2.0 versus before 17.0 ± 2.2, *P* < 0.001).

The difference of the level of IL-17 was negatively correlated with the change of C-ACT score in TCM group and SM group. No correlation was found between the difference of C-ACT score and the levels of IL-10, TGF-*β*1, and MMP-9 in both groups (Tables 6 and 7).

## 4. Discussion

Asthma is a chronic inflammatory disease, in which a variety of cells and cytokines play major roles. Previous studies have indicated that the pathogenesis of airway inflammation in asthma patients may be related to the imbalance of Th1/Th2. Increased number and activation status of Th2 cells cause elevated secretion of Th2-type cytokines (IL-4, IL-5, and IL-10), which are critical factors in the initiation and maintenance of airway inflammation [10, 11]. In previous study, we had found that series empirical prescriptions have a regulatory effect on leukotriene receptor gene expression and the imbalance of Th1/Th2 immune cells in the process of asthmatic attacks [12] and also observed that empirical prescriptions of TCM for the treatment could effectively control asthma attacks and improve multiple lung function indices [6]. To elucidate the immunologic mechanism of TCM in the treatment of asthma, we examined the expression of some more cytokines related to asthma, including IL-10, IL-17, TGF-*β*1, and MMP-9 in this study.

Previously studies showed that the prescription for TCM group, Shegan Mixture, was derived from the Belamcandae and Ephedrine Decoction. Previous study showed that Belamcandae and Ephedrine Decoction can improve the immune function of the patients (effectively preventing hypersensitivity) [13], improve clinical effects in the treatment of pediatric cough-variant asthma, and modify IL-10 and IL-13 serum levels [14]. Luo et al. [15] found that compared with an asthma model group, the Belamcandae and Ephedrine Decoction group showed a reduced expression of TGF-*β*1, decreased thickness of bronchial wall and smooth muscle, and a reduction in inflammatory cells infiltration. In our study, the level of IL-10 decreased after 12 weeks of TCM treatment, suggesting that TCM may inhibit airway inflammation by reducing the secretions of IL-10 to control asthma attacks. We were not able to detect differences following 12 weeks of TCM treatment for TGF-*β*1 serum concentration. The different outcomes of the various studies could be attributed to the different techniques used to quantify the secreted levels of inflammatory mediators.

Recent studies have shown that the imbalance of Th17/Treg is also related to asthma. IL-17 plays an important role in the processes of airway remodeling and airway inflammation by chemotaxis and activation of neutrophils [16]. MMP-9 also participates in airway remodeling by degrading collagen and promoting epithelial cell migration [17]. Miao et al. [18] found that pingchuan mixture can reduce the expression of IL-17 and MMP-9 in lung tissue to improve the process of airway remodeling in asthma. In our study, empirical prescriptions of TCM reduced the levels of IL-17 and MMP-9 in peripheral blood, suggesting that it also has a regulatory effect on the secretion of IL-17 and MMP-9.

At the same time, we also recorded the C-ACT score to assess asthma control. The results showed that the C-ACT scores increased after 12 weeks of TCM treatment, similar to findings of our previous study. Liu et al. [19] found that C-ACT score can reflect the clinical symptoms well, and this conclusion has also been proved in our study. The results also showed that the difference of the level of IL-17 was negatively correlated with the change of C-ACT score in TCM group. We speculate that empirical prescriptions of TCM might inhibit airway inflammation by decreasing the secretion of IL17.

Interestingly, no differences in the levels of IL-10, IL-17, and MMP-9 mRNA were found after treatment in TCM group, with the reduction of those serum levels. These results were somewhat inconsistent with our expectations, so we tried to analyze possible causes of the results. Empirical prescriptions of TCM may affect the levels of cytokine (IL-10, IL-17, and MMP-9) in the process of translation. Besides, the expression process of cytokines is complex, and the number of samples in our study is limited, which may result in the deviation of the results.

The present study demonstrates that empirical prescriptions of TCM can effectively control asthma attacks and reduce the levels of IL-10, IL-17, and MMP-9 in peripheral blood.

## 5. Conclusion

In conclusion, these results demonstrate that empirical prescriptions of TCM have a regulatory effect on the secretion of some inflammatory mediators in pediatric asthma and can help understand the immunologic mechanism of the treatment of asthma.

## Figures and Tables

**Table 1:** Primer sequences and amplicon sizes.

Gene name	Primer sequence (5′ to 3′)	Amplicon size (bp)
GAPDH-F	CTCTCTGCTCCTCCTGTTCGAC	69
GAPDH-R	TGAGCGATGTGGCTCGGCT	
IL10-F	GATGCCTTCAGCAGAGTGAA	105
IL10-F	GCAACCCAGGTAACCCTTAAA	
IL17A-F	GCTGATGGGAACGTGGACTA	125
IL17A-R	CCCACGGACACCAGTATCTT	
TGF*β*-F	TGGTGGAAACCCACAACGAA	113
TGF*β*-R	GAGCAACACGGGTTCAGGTA	
MMP9-F	CATCGTCCACCGGACTCAAA	179
MMP9-R	AAACCGAGTTGGAACCACGA	

**Table 2:** Comparison of the mRNA expression levels of IL-10, IL-17, TGF-*β*1, and MMP-9 between the TCM and SM groups before and after treatment (mean ± SD).

Variables	Before treatment			After treatment		
	TCM group (*n* = 35)	SM group (*n* = 34)	*P* value	TCM group (*n* = 35)	SM group (*n* = 34)	*P* value
IL-17	5.3 ± 8.2	4.2 ± 6.8	0.60	8.0 ± 7.6	8.8 ± 11.2	0.86
TGF-*β*1	196.6 ± 94.9	227.7 ± 130.5	0.32	170.2 ± 83.0	120.3 ± 60.1	0.09
MMP-9	2.5 ± 3.5	1.7 ± 3.6	0.49	2.6 ± 4.5	1.8 ± 3.3	0.66
IL-10	0.5 ± 0.3	0.5 ± 0.5	0.33	0.6 ± 0.6	0.5 ± 0.7	0.73

**Table 3:** Comparison of the mRNA expression levels of IL-10, IL-17, TGF-*β*1, and MMP-9 in the TCM and SM groups before and after treatment (mean ± SD).

Variables	TCM group (*n* = 35)			SM group (*n* = 34)		
	Before	After	*P* value	Before	After	*P* value
IL-17	5.3 ± 8.2	8.0 ± 7.6	0.39	4.2 ± 6.8	8.8 ± 11.2	0.12
TGF-*β*1	196.6 ± 94.9	170.2 ± 83.0	0.44	227.7 ± 130.5	120.3 ± 60.1	0.00
MMP-9	2.5 ± 3.5	2.6 ± 4.5	0.97	1.7 ± 3.6	1.8 ± 3.3	0.89
IL-10	0.5 ± 0.3	0.6 ± 0.6	0.63	0.5 ± 0.5	0.5 ± 0.7	0.63

**Table 4:** Comparison of the peripheral blood levels of IL-10, IL-17, TGF-*β*1, and MMP-9 between the TCM group and SM group before and after treatment (pg/mL, MMP-9 ng/mL, mean ± SD).

Variables	Before treatment			After treatment		
	TCM group (*n* = 35)	SM group (*n* = 34)	*P* value	TCM group (*n* = 35)	SM group (*n* = 34)	*P* value
IL-17	1919.7 ± 1868.4	1845.7 ± 1552.3	0.87	1072.3 ± 689.8	928.1 ± 440.2	0.54
TGF-*β*1	1003.3 ± 914.1	1512.3 ± 997.7	0.02	1079.2 ± 728.1	634.6 ± 391.3	0.07
MMP-9	387.3 ± 119.3	423.1 ± 170.4	0.42	301.4 ± 75.2	331.1 ± 99.3	0.42
IL-10	43.2 ± 61.9	22.6 ± 12.9	0.08	12.4 ± 5.1	16.0 ± 13.2	0.36

**Table 5:** Comparison of the peripheral blood levels of IL-10, IL-17, TGF-*β*1, and MMP-9 in the TCM and SM groups before and after treatment (pg/mL, MMP-9 ng/mL, mean ± SD).

Variables	TCM group (*n* = 35)			SM group (*n* = 34)		
	Before	After	*P* value	Before	After	*P* value
IL-17	1919.7 ± 1868.4	1072.3 ± 689.8	0.04	1845.7 ± 1552.3	928.1 ± 440.2	0.00
TGF-*β*1	1003.3 ± 914.1	1079.2 ± 728.1	0.81	1512.3 ± 997.7	634.6 ± 391.3	0.00
MMP-9	387.3 ± 119.3	301.4 ± 75.2	0.03	423.1 ± 170.4	331.1 ± 99.3	0.03
IL-10	43.2 ± 61.9	12.4 ± 5.1	0.01	22.6 ± 12.9	16.0 ± 13.2	0.13

**Table 6:** Correlation analysis between changes of C-ACT scores and the levels of IL-10, IL-17, TGF-*β*1, and MMP-9 in TCM group.

Variables	d_IL10	d_IL17	d_MMP-9	d_ACT	d_TGF*β*1
d_IL10	1.0000				
d_IL17	−0.9000^*∗*^	1.0000			
d_MMP9	−0.2000	−0.1000	1.0000		
d_C-ACT	0.8721	−0.9747^*∗*^	0.2052	1.0000	
d_TGF*β*1	−0.6000	0.5000	−0.4000	−0.5643	1.0000

^*∗*^ *P* < 0.05.

**Table 7:** Correlation analysis between changes of C-ACT scores and the levels of IL-10, IL-17, TGF-*β*1, and MMP-9 in SM group.

Variables	d_IL10	d_IL17	d_MMP9	d_C-ACT	d_TGF*β*1
d_IL10	1.0000				
d_IL17	0.1190	1.0000			
d_MMP9	0.4762	0.0238	1.0000		
d_C-ACT	−0.4524	−0.7143^*∗*^	−0.2619	1.0000	
d_TGF*β*1	0.4048	0.0000	−0.2381	−0.1429	1.0000

^*∗*^ *P* < 0.05.
